# Risk of atrial fibrillation development in adolescent patients with inflammatory bowel disease

**DOI:** 10.1007/s00431-024-05468-9

**Published:** 2024-02-12

**Authors:** Doaa El Amrousy, Heba El Ashry, Sara Maher, Mohamed Hamza, Samir Hasan

**Affiliations:** 1 Pediatric Department, Faculty of Medicine, Tanta University, Tanta, Egypt; 2 Hepatology and Tropical Medicine Departments, Faculty of Medicine, Tanta University, Tanta, Egypt; 3 Theodor Bilharz Research Institute, Cairo, Egypt

**Keywords:** Inflammatory bowel disease, Atrial fibrillation, Children, Adolescents

## Abstract

There is increasing evidence linking chronic inflammation to the initiation and continuation of atrial fibrillation (AF). Inflammatory bowel diseases (IBD), namely (Crohn’s disease (CD) and ulcerative colitis (UC), are chronic systemic inflammatory disorders with both intestinal and extra-intestinal manifestations. Atrial electromechanical delay (EMD) has been known as an early marker of AF. The objective of this study was to evaluate the atrial electromechanical properties in children and adolescents with IBD during remission. One hundred IBD patients aged 12–17 years (50 with CD and 50 with UC) in remission state and 100 healthy controls were recruited for the study. Atrial electromechanical properties were measured using transthoracic echocardiography, tissue Doppler imaging, and simultaneous surface ECG recording. Interatrial EMD, left intra-atrial, and right intra-atrial EMD were calculated. IBD patients in remission state have significantly prolonged left and right intra-atrial EMD and interatrial EMD compared to healthy controls (*P* = 0.03, *P* = 0.02, and *P* = 0.01 respectively). No statistical difference was observed between CD and UC in terms of inter- and intra-atrial EMDs.

*Conclusion*: Atrial EMD is increased in pediatric patients with IBD indicating the increased risk of AF development. Measurement of atrial EMD parameters might be used to predict the risk of the development of AF in pediatric patients with IBD.

**Table:** 

**What is Known:** *• There is increasing evidence linking chronic inflammation to the initiation and continuation of atrial fibrillation (AF). * *• Inflammatory bowel diseases are chronic systemic inflammatory disorders with both intestinal and extra-intestinal manifestations.* *• Atrial electromechanical delay (EMD) has been reported as an early marker of AF.*	
**What is New:** *• Atrial EMD is increased in pediatric patients with IBD indicating the increased risk of AF development.* *• Measurement of atrial EMD parameters might be used to predict the risk of the development of AF in pediatric patients with IBD.*

**What is Known:**

*• There is increasing evidence linking chronic inflammation to the initiation and continuation of atrial fibrillation (AF). *

*• Inflammatory bowel diseases are chronic systemic inflammatory disorders with both intestinal and extra-intestinal manifestations.*

*• Atrial electromechanical delay (EMD) has been reported as an early marker of AF.*

**What is New:**

*• Atrial EMD is increased in pediatric patients with IBD indicating the increased risk of AF development.*

*• Measurement of atrial EMD parameters might be used to predict the risk of the development of AF in pediatric patients with IBD.*

## Introduction

Inflammatory bowel diseases (IBD), which include both Crohn’s disease (CD) and ulcerative colitis (UC), are chronic, heterogeneous, relapsing inflammatory disorders of the gastrointestinal system with multiple extra-intestinal manifestations that severely affect the growth, development, and general health of children [1]. Recently, the incidence of IBD increased globally, both in children and adults, and more than 35% of adults with IBD started their symptoms before the age of 18 years [2].

IBD patients have an increased risk of cardiovascular diseases (CVDs). The relationship between CVDs such as stroke, coronary artery disease, and IBD has been reported in adults [3]. Death from CVDs is increased in patients with IBD compared with the normal population [4]. Although ischemic heart disease and stroke are the main reasons in IBD patients, several cardiac complications could also occur in these patients such as pericarditis, myocarditis, and thromboembolic events [5, 6]. There is limited data about ventricular events or electrical abnormalities in patients with IBD [7]. Curione et al. [8] demonstrated that QTc interval and QT dispersion were increased in patients with IBD.

Atrial fibrillation (AF) is one of the most common cardiac arrhythmias associated with a significant increase in mortality and morbidity risk from stroke [9]. Recently, inflammation has been shown to be a pathogenic factor contributing to the development of AF [10]. The relationship between inflammation and AF is not completely understood; however, atrial myocarditis that develops as a result of infiltration of the atrial wall with inflammatory cells is thought to be an etiologic factor [11]. As AF is known to have fatal complications such as stroke, it will be essential to predict the disease in advance to prevent complications [12]. It may be a rational approach to evaluate atrial conduction times (ACTs) to determine the risk before arrhythmia develops.

There was very limited data about AF and IBD in children, and (up to our knowledge) this is the first research to evaluate the link between AF and IBD in such population.

## Methods

This prospective cross-sectional study was carried out at the pediatric and gastroenterology departments, Tanta University Hospital between June 2022 and May 2023. One hundred consecutive IBD pediatric patients aged 12–17 years old were included as the patient group. All included patients were previously diagnosed as either to be CD or UC patients at least 2 years before enrollment depending on history, laboratory parameters, and endoscopic biopsies. All included patients were in remission depending on pediatric Crohn’s disease activity index (PCDAI) and pediatric ulcerative colitis activity index (PUCAI) < 10 for at least 3 months before enrollment. The control group included 100 healthy children who attended our outpatient clinics for minor complaints and were matched for age and sex.

Exclusion criteria were as follows: subjects with known congenital or acquired heart disease, bundle branch block in the electrocardiogram (ECG), anti-arrhythmic drug use for any reason, active infection, diabetes mellitus (DM), Cushing syndrome, hypertension, hypothyroidism or hyperthyroidism, and patients who had previously been diagnosed with supra-ventricular tachycardia.

This study was conducted in accordance with the principles of the Declaration of Helsinki, and it was approved by the ethical committee of our faculty of Medicine. At the start of the study, all subjects and their parents were fully informed about the study, their rights, and objectives of our study and signed a written informed consent after approval to participate in the study.

The study groups were evaluated with a complete physical examination, 12-lead ECG, standard transthoracic echocardiography, and tissue Doppler imaging (TDI). Body mass index (BMI) was calculated by dividing weight by the square of height (BMI = weight/(height in m^2^). Systolic and diastolic blood pressure was also measured.

### Electrocardiography (ECG) analysis

After 10 min of rest, 12-lead ECG recordings were obtained on paper with each subject in the supine position at a size of 10 mm/mv and a speed of 50 mm/s [13]. CardioLab v. 6.0 General Electric Medical SystemsTM was used for ECG recordings and measurements. ECGs were analyzed by two experts without any knowledge of the patients’ clinical conditions.

### Standard echocardiography and Doppler measurements

Transthoracic echocardiographic examinations were performed by experienced echocardiographers, who were blinded to the study groups, using the Vivid 7^®^ cardiac ultrasonography system (GE Ving-Med Ultrasound AS; Horten, Norway) with 2.5- to 5-MHz probes. M-mode, pulsed, and color flow Doppler echocardiography. A single lead electrocardiogram is continuously recorded. The examinations were performed in accordance with the recommendations of the American Society of Echocardiography. All conventional measurements such as left ventricular ejection fraction (LV EF), left ventricular end-systolic diameter (LVESD), left ventricular end-diastolic diameter (LVEDD), inter-ventricular septum thickness (IVS), posterior wall thickness (PW), aortic root diameter (AR), and ascending aortic root diameter (AAR) were performed. Left anteroposterior atrial diameter (LA) in the parasternal long-axis view at the end of ventricular systole was also measured. LV EF was calculated by Simpson’s method [14]. Transmitral pulsed-wave flow velocities were measured by using the apical four-chamber view. Early (E) and late (A) diastolic peak flow velocities and their ratio (E/A) were also measured.

### Tissue Doppler imaging (TDI)

TDI imaging technique was used to measure atrial electromechanical association. TDI provides analysis of high-amplitude and low-speed movements of the myocardium from different regions of the heart with high temporal resolution. The sample volume width is kept in the range of 2–5 mm in order to achieve high temporal resolution. Due to the low myocardial velocities, measurements were done when Nyquist limits were between –20 and +20 cm/s and the monitor speed was between 50 and 100 mm/s. When the pulsed Doppler sample volume was on the apical four-chamber view axis, it was placed in the left ventricle lateral mitral annulus, septal mitral annulus, and right ventricle tricuspid annulus. The sampled window was positioned as parallel as possible to the myocardial area from which an optimal view was desired. Inter-atrial and intra-atrial electromechanical delay (EMD) durations were determined using TDI and ECG tracing simultaneously. The time between the onset of the P wave on the ECG and the beginning of the tissue Doppler late diastolic wave was defined as the atrial electromechanical union (PA) time. Inter-atrial EMD (IA-EMD) was calculated as the time difference between lateral PA and tricuspid PA. The time difference between septal PA and tricuspid PA was calculated as intra-right atrial EMD (RI-EMD) and the time difference between lateral PA and septal PA was calculated as intra-left EMD (LI-EMD).

### Laboratory investigations

Routine laboratory investigations such as serum cholesterol, triglycerides, fasting blood glucose, serum urea and creatinine, and liver function test were performed.

Inflammatory markers such as high-sensitivity C-reactive protein (hs-CRP), erythrocyte sedimentation rate (ESR), interleukin-10 (IL-10), IL-12, IL-17, IL-23, and tumor necrosis factor alpha (TNF-α) were also evaluated.

### Statistical analysis

All quantitative data were expressed as mean ± standard deviation or median and range according to the normality of the data. The data were tested for normal distribution by the Kolmogorov-Smirnov test. A categorical variable was expressed using a percentage. Student’s *t*-test was used in cases of a normal distribution of values, and the Mann-Whitney *U*-test was used in cases that did not comply with the normal distribution. Differences between the means of more than two groups were analyzed by one-way analysis of variance (ANOVA) followed by post-hoc analysis. Spearman correlation analysis was used to evaluate the relationship between electromechanical and clinical parameters. All the analyses were conducted using SPSS V.20 (SPSS, Chicago, IL, USA). The statistical significance level was considered if *P* < 0.05.

## Results

The basic demographic, clinical, and laboratory data of participants are summarized in Table 1. There was no significant difference between IBD patients and the control group regarding age, sex, BMI, systolic and diastolic blood pressures, fasting blood glucose level, serum triglycerides, serum cholesterol, serum creatinine, and liver enzymes. All measured inflammatory markers (hs-CRP, ESR, IL-17, IL-10, IL-12, IL-23, and TNF-α) were significantly higher in IBD patients compared to the control group.

**Table 1:** Demographic clinical and laboratory data of the study population

**Variables**	**Control (*n* = 100)**	**IBD (*n* = 100)**	***P***
Age (y)	15.6 ± 1.8	16.1 ± 1.9	0.63
Sex (male to female)	51:49	52:48	0.82
SBP (mm/Hg)	122.3 ± 12.5	124.2 ± 11.9	0.35
DBP (mm/Hg)	72 (66–81)	77 (69–92)	0.1
BMI (kg/m^2^)	25.1 (17.3–33.5)	24.1 (16.9–38.2)	0.12
Illness duration (y)	-	2.9(2.1–4.1)	-
Fasting glucose (mg/dl)	88.2 ± 7.4	92.5 ± 10.1	0.4
Triglycerides (mg/dl)	121.7 ± 22.0	118.9 ± 25.8	0.64
Cholesterol (mg/dl)	134.3 ± 15.8	142.1 ± 20.4	0.35
ESR (mm/h)	7.1 (2–14)	12.6(2–29)	**0.01**
hs-CRP (mg/l)	1.05 (0.1–9.0)	8.2(0.1–17)	**0.03**
Creatinine (mg/dl)	0.8 (0.4–1.1)	0.8(0.5–1.1)	0.7
AST (U/L)	17.5 ± 6.2	18.5 ± 6.4	0.1
ALT (U/L)	16.3 ± 7.5	19.8 ± 11.3	0.14
TNF-α (pg/ml)	16.3 ± 3.8	21.2 ± 5.9	**0.02**
IL-17 (pg/ml)	8.75 ± 3.13	14.1 ± 5.3	**0.001**
IL-10 (pg/ml)	7.9 ± 1.3	9.2 ± 3.5	**0.03**
IL-12 (pg/ml)	58.4 ± 24.2	72.3 ± 28.6	**0.01**
IL-23 (pg/ml)	45.1 ± 18.5	60.7 ± 19.6	**0.02**

Data in bold emphasis indicate significant difference

*SBP* systolic blood pressure, *DBP* diastolic blood pressure, *BMI* body mass index, *ESR* erythrocyte sedimentation rate, *hs-CRP* high sensitivity C-reactive protein, *AST* aspartate aminotransferase, *ALT* alanine aminotransferase, *TNF-α* tumor necrosis factor alpha, *IL* interleukin

Table 2 shows disease locations according to the Montreal classification. In the CD group, three patients (6%) had upper GIT location (L4), 20 patients (40%) in the ileal region (L1), 17 patients (34%) in the illiocolonic region (L3), and 10 patients (20%) in the colon (L2).

**Table 2:** Disease location (with Montreal classification) and patient’s treatment

Variable	CD (*n* = 50)	UC (*n* = 50)	
Disease location, *n* (%), classification	Upper GIT, 3 (6%) L4	Proctitis, 32 (64%) E1	
	Ileal, 20 (40%) L1	Left-sided colitis, 10 (20%) E2	
	Iliocolonic, 1 7 (34%) L3	Extensive colitis, 8 (16%) E3	
	Colonic, 10 (20%) L2		
Treatment, *n* (%)			*P*
1-Aminosallicylate	-	8 (16)	
2-Corticosteroids	15 (30)	11 (22)	
3-Azathioprine	24 (46)	17 (34)	0.72
4-Biological	8 (16)	9 (18)	
5-Combination	5 (10)	7 (14)	

*UC* ulcerative colitis, *CD* Crohn’s disease

*Montreal classification: L1-L4 for CD, E1-E3 for UC

In patients with UC, 32 patients (64%) had proctitis (E1). Ten patients (20%) had left-sided colitis (E2), and eight patients (16%) had extensive colitis (E3). There was no significant difference between CD patients and UC patients regarding treatment drugs.

Echocardiographic characteristics and atrial electromechanical parameters of the study participants are shown in Table 3. There was a non-significant difference between IBD patients and the control group regarding LVEF, left atrium diameter, IVS, deceleration time, early diastolic wave, late diastolic wave, heart rate, and tricuspid PA. However, the atrial electromechanical parameters measured showed that left intra-atrial EMD, right intra-atrial EMD, and inter-atrial EMD were significantly longer in the IBD patients compared to the healthy controls. PA (time interval from the onset of the P-wave on the surface electrocardiogram to the beginning of the late diastolic wave) lateral and septal times were significantly longer in the IBD patients compared to the healthy controls.

**Table 3:** Echocardiographic characteristics and atrial electromechanical parameters of the study participants

Variable	Control (*n* = 100)	IBD (*n* = 100)	*P*^1^	CD (*n* = 50)	*P*^2^	UC (*n* = 50)	*P*^3^
LVEF (%)	63.7 ± 3.4	62.5 ± 3.1	0.07	62.1 ± 2.9	0.09	62.8 ± 3.0	0.06
LA AP diameter (cm)	3.2 (2.7–3.6)	3.3 (2.7–4.0)	0.7	3.4 (2.8–4.1)	0.81	3.2 (2.7–3.9)	0.66
IV Septal wall (cm)	0.87 ± 0.11	0.94 ± 0.51	0.82	0.93 ± 0.31	0.91	0.97 ± 0.62	0.73
PW thickness (cm)	0.84 ± 0.12	0.96 ± 0.44	**0.04**	0.94 ± 0.68	**0.04**	0.97 ± 0.61	**0.02**
Deceleration time (ms)	189.4 ± 27.5	197.4 ± 57.2	0.62	194.1 ± 62.7	0.68	201.4 ± 66.8	0.61
E/Em	7.5 ± 1.62	7.8 ± 2.1	0.81	7.9 ± 2.0	0.9	7.8 ± 2.3	0.77
E	80.4 ± 9.2	77.3 ± 18.4	0.59	76.8 ± 20.3	0.72	76.1 ± 19.9	0.55
A	71.3 ± 14.8	69.6 ± 24.1	0.24	69.1 ± 22.8	0.51	76.8 ± 25.3	0.13
HR (beat/min)	75.2 ± 11.0	74.2 ± 10.7	0.78	73.9 ± 11.2	0.79	72.5 ± 11.7	0.25
PA lateral (ms)	55.1 ± 6.8	63.9 ± 8.3	**< 0.001**	63.1 ± 9.2	**< 0.001**	61.4 ± 9.0	**< 0.001**
PA septal (ms)	38.6 ± 5.7	42.3 ± 7.5	**0.01**	41.0 ± 7.9	**0.02**	42.9 ± 8.2	**0.01**
PA tricuspid (ms)	31.7 ± 4.9	32.3 ± 3.7	0.46	32.1 ± 4.1	0.37	33.1 ± 5.2	0.49
IA-EMD (ms)	21.92 ± 5.88	30.7 ± 8.5	**< 0.001**	30.1 ± 9.2	**< 0.001**	31.1 ± 9.4	**< 0.001**
RI-EMD (ms)	6.62 ± 2.41	10.8 ± 5.8	**0.02**	10.2 ± 6.0	**0.02**	11.2 ± 6.4	**0.01**
LI-EMD (ms)	14.8 ± 3.9	19.5 ± 7.3	**0.03**	19.1 ± 8.2	**0.04**	20.1 ± 8.5	**0.02**

Data in bold emphasis indicate significant difference

*P*^1^ for comparison between IBD patients and the control group, *P*^2^ for comparison between CD patients and the control group, *P*^3^ for comparison between UC patients and the control group

*IBD* inflammatory bowel disease, *CD* Crohn’s disease, *UC* ulcerative colitis, *LVEF* left ventricular ejection fraction, *LA* left atrium, *AP* anteroposterior, *PW* posterior wall, *E* early diastolic wave, *A* late diastolic wave, *Em* tissue Doppler-derived early diastolic septal wave, *HR* heart rate, *PA* time interval from the onset of the P-wave on surface electrocardiogram to the beginning of the late diastolic wave, *IA-EMD* interatrial electromechanical delay, *RI-EMD* right intra-atrial electromechanical delay, *LI-EMD* left intra-atrial electromechanical delay

Correlation analysis between inflammatory markers and echocardiographic and electromechanical parameters (Table 4) showed that there were significant positive correlations between both ESR and hs-CRP with all measured echocardiographic parameters except LVEF, early diastolic wave (E), and E/A ratio which showed significant negative correlations.

**Table 4:** Correlation between inflammatory markers (ESR and hs-CRP) with echocardiographic findings and electromechanical parameters

Variables	ESR		hs-CRP	
	*r*	*P*	*r*	*P*
LVEF (%)	−0.35	**0.02**	−0.57	**0.04**
LA (cm)	0.66	**< 0.001**	0.27	**< 0.001**
IV Septal wall (cm)	0.46	**0.01**	0.39	**0.01**
PW thickness (cm)	0.14	**< 0.001**	0.15	**< 0.001**
E/Em	0.61	**0.04**	0.58	**0.03**
E	−0.35	**0.01**	−0.29	**0.01**
A	0.36	**0.01**	0.47	**0.02**
E/A	−0.18	**< 0.001**	−0.19	**< 0.001**
PA lateral (ms)	0.49	**< 0.001**	0.36	**< 0.001**
PA septal (ms)	0.51	**< 0.001**	0.62	**< 0.001**
PA tricuspid (ms)	0.46	**< 0.001**	0.52	**< 0.001**
IA-EMD (ms)	0.50	**< 0.001**	0.37	**< 0.001**
RI-EMD (ms)	0.33	**< 0.001**	0.46	**< 0.001**
LI-EMD (ms)	0.39	**0.02**	0.27	**0.01**

Data in bold emphasis indicate significant difference

*LVEF* left ventricular ejection fraction, *LA* left atrium, *PW* posterior wall, *E* early diastolic wave, *A* late diastolic wave, *Em* tissue Doppler-derived early diastolic septal wave, *HR* heart rate, *PA* time interval from the onset of the P-wave on surface electrocardiogram to the beginning of the late diastolic wave, *IA-EMD* interatrial electromechanical delay, *RI-EMD* right intra-atrial electromechanical delay, *LI-EMD* left intra-atrial electromechanical delay

## Discussion

Considering that the pathogenesis of AF is being increasingly linked to systemic inflammation, IBD may be a potential risk factor for AF. However, there is limited information regarding the association between the risk of developing AF and the presence of IBD. Studies of the cardiovascular risk in patients with IBD have focused on venous and arterial thrombotic events [15, 16], whereas to our knowledge, pediatric studies of AF risk in IBD have not previously been reported.

AF is the most common sustained cardiac arrhythmia and is associated with increased hospitalization, decreased quality of life, and exercise capacity. Affected individuals may have a fivefold risk of stroke, a threefold risk of heart failure (HF), and a twofold increased risk of mortality [17, 18]. According to these deleterious effects, it is important to determine the susceptibility to AF, and thus, some noninvasive methods have been described to predict the development and recurrence of AF in several systemic diseases. Atrial conduction time (ACT) represents atrial depolarization time between the sinus node and the farthest points of atria. Prolonged interatrial and intra-atrial conduction time, namely EMD, was shown to be related to the development and recurrence of AF in patients with or without heart disease [18, 19]. EMD was shown to be a predictor of new-onset AF, recurrence of AF after cardioversion, and new-onset HF after myocardial infarction [20, 21]. Moreover, Chao et al. [22] demonstrated that the atrial electromechanical interval is associated with atrial remodeling that finally leads to AF. Two mechanisms have been suggested to elucidate the EMD and AF association: prolonged conduction time-induced daughter wavelets in different parts of the atria and/or prolonged conduction time facilities reentry, both of which finally initiate AF [23, 24]. Electrophysiology is the appropriate method for the measurement of ACT, but it is invasive and difficult to use in daily practice. A combined electro-echocardiographic method has been developed to assess ACT, and hence, it provides noninvasive detection of the individuals who are at risk of AF [25].

In our study, we demonstrated that both interatrial and intra-atrial conduction times were significantly prolonged in children and adolescents with IBD compared to healthy controls. Limited studies in adults demonstrated conflicting results. An increased risk of AF during active stages of IBD, with no increased risk observed in remission periods, was reported in one study [26]. Others recorded higher risk during all stages of IBD with greater risk in patients with active disease compared to those in remission state [27], last recorded a higher risk of AF in IBD patients during remission state compared to healthy controls [27, 28].

A number of studies have linked systemic inflammation, namely elevated levels of CRP, with an increased risk of AF [29]. Moreover, a role of inflammation in the pathogenesis of AF has been suggested by the reduction of AF by corticosteroid treatment in post-cardiac surgery and the finding that AF in itself may give rise to atrial inflammation and promote electrical remodeling favoring AF [11].

In our study, IBD patients in the remission state showed significantly higher hs-CRP and ESR values and a positive correlation with EMD. This means that increased inflammatory load, represented by increased hs-CRP and ESR, could be the cause of EMD. Efe et al. [27] demonstrated prolonged EMD in IBD patients with remission compared to the control; however, they did not find any significant differences in terms of hs-CRP and ESR and they thought that these parameters do not reflect the current inflammation, so the inflammatory load may still be high and the inflammatory changes have already occurred in various tissues like the atrium.

The chronic inflammatory conditions found in IBD are the key element in the pathogenesis of arrhythmias. The chronic inflammatory process mediated by pro-inflammatory cytokines (CRP, IL-6, and TNF-α) causes interstitial fibrosis and impairs the intracellular calcium current resulting in structural and electrical remodeling that results in arrhythmias [30]. Moreover, chronic inflammation also leads to the occurrence of autonomic dysregulation (increased sympathetic tone and decreased parasympathetic tone) resulting in reduced heart rate variability and prolonged QT interval which contribute to the development of arrhythmias [31].

## Limitations

Cross-sectional design and lack of follow-up in terms of future arrhythmic episodes, in order to see whether patients with EMD develop AF (especially during the active stage), are the major limitations of our study. We also did not assess the presence or absence of atrial fibrous tissue by other imaging modalities such as magnetic resonance imaging. However, this study may pave the way for other broader research in the future for a deeper and more accurate evaluation of the arrhythmogenic potential of IBD disease in young people.

## Conclusions

Atrial EMD increased in adolescents with IBD during the remission state. Therefore, we need to consider screening for AF, as timely intervention can reduce both morbidity and mortality secondary to the complications of AF.

## Data Availability

The dataset used and/or analyzed during the current study is available from the corresponding author on reasonable request.
